# Novel Cellulosic Fiber Composites with Integrated Multi-Band Electromagnetic Interference Shielding and Energy Storage Functionalities

**DOI:** 10.1007/s40820-025-01652-0

**Published:** 2025-01-31

**Authors:** Xuewen Han, Cheng Hao, Yukang Peng, Han Yu, Tao Zhang, Haonan Zhang, Kaiwen Chen, Heyu Chen, Zhenxing Wang, Ning Yan, Junwen Pu

**Affiliations:** 1 Beijing Key Laboratory of Lignocellulosic Chemistry, College of Materials Science and Technology, Beijing Forestry University, Beijing, 100083 People’s Republic of China; 2 Department of Chemical Engineering and Applied Chemistry, University of Toronto, Toronto, ON M5S3E5 Canada; 3 Key Laboratory of Advanced Marine Materials, Ningbo Institute of Materials Technology and Engineering, Chinese Academy of Sciences, Ningbo, 315201 People’s Republic of China; 4 College of Mechanical and Electronic Engineering, Northwest A&F University, Yangling, 712100 Shaanxi People’s Republic of China; 5 Jiangsu Provincial Key Lab of Sustainable Pulp and Paper Technology and Biomass Materials, Nanjing Forestry University, Nanjing, 210037 People’s Republic of China; 6 Co-Innovation Center of Efficient Processing and Utilization of Forest Resources, Nanjing Forestry University, Nanjing, 210037 People’s Republic of China

**Keywords:** Cellulose fiber skeleton, TEMPO mediated oxidation, In-situ polymerization, Multi-band EMI shielding, Energy storage

## Abstract

In an era where technological advancement and sustainability converge, developing renewable materials with multifunctional integration is increasingly in demand. This study filled a crucial gap by integrating energy storage, multi-band electromagnetic interference (EMI) shielding, and structural design into bio-based materials. Specifically, conductive polymer layers were formed within the 2,2,6,6-tetramethylpiperidine-1-oxide (TEMPO)-oxidized cellulose fiber skeleton, where a mild TEMPO-mediated oxidation system was applied to endow it with abundant macropores that could be utilized as active sites (specific surface area of 105.6 m^2^ g^−1^). Benefiting from the special hierarchical porous structure of the material, the constructed cellulose fiber-derived composites can realize high areal-specific capacitance of 12.44 F cm^−2^ at 5 mA cm^−2^ and areal energy density of 3.99 mWh cm^−2^ (2005 mW cm^−2^) with an excellent stability of maintaining 90.23% after 10,000 cycles at 50 mA cm^−2^. Meanwhile, the composites showed a high electrical conductivity of 877.19 S m^−1^ and excellent EMI efficiency (> 99.99%) in multiple wavelength bands. The composite material’s EMI values exceed 100 dB across the L, S, C, and X bands, effectively shielding electromagnetic waves in daily life. The proposed strategy paves the way for utilizing bio-based materials in applications like energy storage and EMI shielding, contributing to a more sustainable future.

**Supplementary Information:**

The online version contains supplementary material available at 10.1007/s40820-025-01652-0.

## Highlights

- A mild 2,2,6,6-tetramethylpiperidine-1-oxide mediated modification system was applied to improve the reactivity and introduce porous structure of cellulose fiber skeleton.
- Composite exhibited highly efficient electromagnetic shielding interference performance (> 99.99%) over multi-band.
- Integrating energy storage, electromagnetic interference shielding, and structural design into bio-based materials.

**Supplementary Information:**

The online version contains supplementary material available at 10.1007/s40820-025-01652-0.

## Graphical abstract

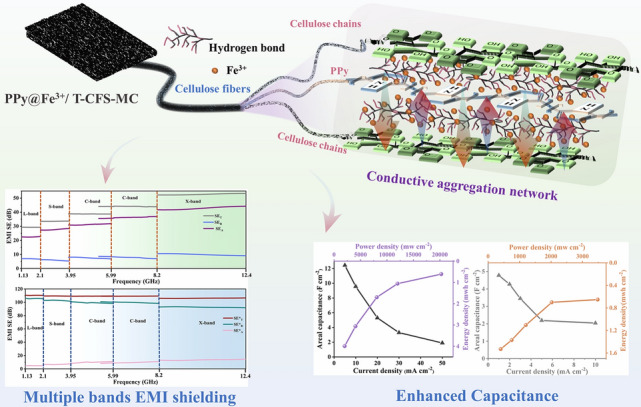

## Introduction

In recent years, there are increased interests in utilizing cellulosic fiber-based materials for application in energy storage devices [1–4]. Owing to the abundant functional groups and active sites on the surface of cellulose fibers, this type of energy storage devices exhibit fast charging and discharging capabilities, high power density, and robust cycle stability [5, 6]. To improve the energy storage performance of cellulosic fiber-based materials, the inherent three-dimensional framework structure derived from natural wood was utilized. Cellulose fiber skeleton (CFS) is prepared first via the delignification of wood to improve the accessibility of the wood cell wall and expose cellulose on the surface. 2,2,6,6-Tetramethylpiperidine-1-oxide (TEMPO) oxidation treatment is a common technique to further improve the reactivity of cellulose [7–9] that is used to enhance energy storage performance [10]. This modification method can also improve the surface charge density of the fiber [11]. However, in the conventional TEMPO/NaClO/NaBr modification system (pH = 10) [12] [13, 14], C-6 aldehyde produced by TEMPO/NaClO oxidation under alkaline conditions leads to the depolymerization of cellulose, resulting in a loss of fiber mechanical performance [15, 16]. Therefore, a mild TEMPO/NaClO/NaClO_2_ (pH = 7) modification system has been developed, where the C6 aldehyde generated by TEMPO/NaClO oxidation is further oxidized by NaClO_2_ under neutral conditions to prevent extensive cellulose depolymerization [11, 17]. Nevertheless, the production of cellulose-based energy storage devices often requires harsh processing conditions, such as carbonization and etching, to achieve high performance. This undoubtedly increases costs and significantly limits the commercial application of these devices [18, 19].

To simplify the fabrication of cellulose-based energy storage materials, intrinsic conductive polymers, like polypyrrole (PPy), polyanilines (PANI), and polythiophene (PT), have emerged as potential alternatives to carbon-based materials in recent years [20, 21]. These materials offer comparable conductivity and high pseudo-capacitance, and their straightforward preparation process allows for readily polymerization with cellulose fibers [22–25]. For instance, Dong et al. synthesized a LiFe_5_O_8_@PPy core–shell nanocomposite electrode material with greatly improved electrochemical performance and cyclic stability with capacitance maintained at 82.99% after 10,000 times charging and discharging cycles [26]. Although the presence of conductive polymers can significantly improve the electrochemical performance of the cellulose materials, they failed to form a stable charge transfer layer within the cellulose network, and unable to achieve a theoretically high capacitance. Hence, an efficient processing strategy is needed to construct a stable fiber-conductive polymer charge transfer layer that is compatible with the wood fiber structure to fully realize the potential of cellulose fiber-based materials.

Moreover, the increasing use of electronic devices in daily life leads to pervasive electromagnetic radiation, which not only negatively impacts the normal operation of equipment but also poses potential risks to human health [27, 28]. As a result, there is a growing demand for effective electromagnetic interference (EMI) shielding materials to mitigate these problems. EMI shielding refers to the technique of blocking or reducing the electromagnetic field within a space by barriers using conductive or magnetic materials [29–31]. Electromagnetic waves (EMWs) constitute a form of electromagnetic radiation characterized by their wave nature and broad frequency spectrum. Microwaves, which are widely used in modern technology, are categorized into L-band (1–2 GHz), S-band (2–4 GHz), C-band (4–8 GHz), and X-band (8–12 GHz) [32–35]. While most studies report EMI shielding performance primarily in the X-band, EMWs generated by commonly used devices like laptops (2.4 GHz, 5 GHz), mobile phones (1.7–2.6 GHz), and Wi-Fi routers (2.4 GHz, 5 GHz) are concentrated in other frequency bands. Therefore, developing materials with EMI shielding capacities across multiple frequency bands is beneficial for achieving comprehensive protection. However, achieving this multi-band shielding presents significant challenges, as the mechanism for shielding varies across different frequency ranges. In the low-frequency bands (L and S bands), where the wavelength of EMWs is longer, shielding is predominantly relied on conductive and magnetic loss. The size of the material usually needs to be comparable to or larger than the wavelength to prevent leakage from the edge (boundary effect) [36]. In contrast, high-frequency bands (C and X bands) involve shorter wavelength (~ 7.5–2.5 cm), and shielding mainly relies on dielectric loss, absorption and scattering of electromagnetic waves. To achieve shielding in these higher frequencies, it is necessary to possess optimized microstructures, such as hierarchical pores, increased surface area, and enhanced conductivities [37]. These structural requirements add complexity to the material design process. Therefore, to achieve effective multi-band EMI shielding, it is essential to introduce functional components (e.g., magnetic materials, high dielectric constant materials), and optimize both the microstructure and multi-scale interface design.

Furthermore, many electronic and electrical devices generate electromagnetic waves that can interfere with the performance of energy storage systems, reducing their operational life and efficiency [36]. Therefore, there is a pressing need to develop multifunctional materials that can provide both high-performance EMI shielding and energy storage capabilities. Integrating these functionalities within a single material system represents a significant innovation, as it addresses both the growing demand for energy storage and the need for protection against electromagnetic interference.

In this study, a cellulosic fiber-based composite with enhanced capacitance and multi-band EMI shielding performance was fabricated. Specifically, wood specimen was delignified to obtain CFS, which possessed a unique 3D framework porous structure, and a high specific surface area. Then CFS was subjected to a mild TEMPO oxidation to obtain the modified cellulose fiber that had a multi-layered interconnected porous structure with more active sites on the fiber surface in addition to the high specific surface area of the materials. Finally, the pyrrole monomer was in situ polymerized with Fe^3+^ on the modified fiber structure to form a stable charge transfer layer to construct the polypyrrole (PPy)@Fe^3+^-TEMPO cellulose fiber multifunctional composites (PPy@Fe^3+^/T-CFS-MC). The new materials exhibited a high areal specific capacitance of about 12.44 F cm^−2^ at 5 mA cm^−2^ and a high areal energy density of 3.99 mWh cm^−2^ with an excellent stability of 90.23% after 10,000 cycles at 50 mA cm^−2^. Moreover, the material achieved outstanding multi-band electromagnetic shielding performance through multi-scale interface structural design (CFS), microstructure optimization (TEMPO oxidation), and the incorporation of magnetic components (Fe^3+^) and conductive polymers (PPy). The EMI values in the L, S, C, and X bands were over 100 dB. The novel high-performance, multi-band electromagnetic radiation-resistant cellulosic fiber-based composite material has excellent promise for applications in a broad range of energy storage devices.

## Experimental Section

### Materials

Balsa wood samples were purchased from Shan Dong Province, China. Sodium chlorite (NaClO_2_, AR), sodium hydroxide (NaOH, AR), Pyrrole (Py, AR), ferric chloride hexahydrate (FeCl_3_·6H_2_O, AR), glacial acetic acid (CH_3_COOH, AR), sodium sulfate (Na_2_SO_4_, AR), sodium bromide (NaBr, AR), TEMPO (2,2,6,6-tetramethylpiperidine-1-oxyl, AR) and sodium hypochlorite (NaClO, AR) were provided from Aladdin Chemistry Co., Ltd, China. The solvent is deionized (DI) water.

### Preparation of the Cellulose Fiber Skeleton

The wood samples were sawed into specific sizes. According to our previous work, the CFS was prepared from wood samples [38]. In short, the wood samples were put into prepared 4% NaClO_2_ solution and adjusted to a pH value of 4.0 with CH_3_COOH at 85 °C for 8–24 h (wood samples of different sizes have different reaction times). Subsequently, the treated wood samples were rinsed with DI water and then freeze-dried to obtain the CFS.

### Preparation of TEMPO Oxidation the CFS

The CFS (dry mass of 1 g) was soaked in 200 mL of DI water containing 0.032 g TEMPO and 2.26 g NaClO_2_, and then adjusted a pH value of 7.0 with CH_3_COOH solution. 20 mL NaClO solution (8 wt% NaClO) was added to the beaker to initiate the oxidation reaction. The pH value of the solution was closely monitored using a pH meter and was maintained at 7 by continuously adding CH_3_COOH solution. The reaction was carried out at 60 °C for 48 h without stirring to avoid mechanical disintegration. Finally, the treated CFS were rinsed with DI water and then frozen with liquid nitrogen. Then, the samples were freeze-dried to prepare composites, named T-CFS.

### Preparation of Multifunctional Composites

First, T-CFS were immersed in the beaker prepared with Pyrrole. The beaker was then placed in a vacuum drying oven for vacuum degassing for 10 min. Following degassing, in situ polymerization of the samples was performed by immersing the samples in 1 M FeCl_3_·6H_2_O solution for 24 h. Subsequently, the samples were taken out and rinsed with DI water to remove residual reagents. Finally, the samples were naturally air-dried and named as PPy@Fe^3+^/T-CFS-MC.

### Characterization

The micromorphology of NW and CFS was evaluated by scanning electron microscope (SEM, ZEISS Gemini 300, Germany). SEM was used to analyze the micromorphology and elemental patterns of the lyophilized PPy@Fe^3+^/T-CFS-MC. The samples were pretreated at 120 °C in vacuum for 12 h, and then N_2_ adsorption/desorption was detected by four-station automatic surface area analyzer (Micromeritics APSP 2460, USA) under 77 K liquid nitrogen condition. After the instrument analysis was completed, the isothermal adsorption/desorption curve was obtained, and the total specific surface area of the materials was obtained according to Brunauer-Emmet-Teller (BET) method. The samples were subjected to functional group analysis by Fourier transform infrared spectroscopy (ATR-FTIR, Thermo Scientific, USA). All samples were tested using cumulative 32 scans with a resolution of 4.00 cm^−1^ at 600–4000 cm^−1^. X-ray diffraction (XRD, Ultima IV, Japan) at 3 kW with Cu-Kα (λ = 1.5406 Å) radiation in the testing range of 10°–80° was used to investigate crystalline regions of the samples. X-ray photoelectron spectrometer (XPS, Thermo Scientific, USA) results were detected on an AMICUS spectrometer with X-ray source Al Kα.

#### Electrical Conductivity and Electromagnetic Shielding Performance Measurements

The electrical conductivity of samples was measured with the aid of an electronic multimeter (UT890C, UNI-T, China). The size of the prepared samples was 10 × 10 × 1 mm, and the average value of the five tests was taken. The calculation formula of resistivity (ρ) is shown below:

1 $$ \rho = {\text{RS}}/{\text{L}} $$

According to Ohm’s law, conductivity (G) is the reciprocal of resistivity (ρ), which is determined by voltage and current.

2 $$ {\text{G}} = {1}/\rho $$

where ρ (Ω m) is the resistivity, *S* (m^2^) is the cross-sectional area, *R* (Ω) is the resistance value, *L* (m) is the length of the wire, *G* (S m^−1^) is conductivity.

The EMI shielding effectiveness (EMI SE) of the samples was measured at room temperature using a vector network analyzer (Agilent PNA N5244A, USA) based on the waveguide method. In our setup, the adapter fixture and the vector network analyzer were first connected. Next, opened the testing software, set the frequency to the desired range, and performed two-port calibration. After calibration, aligned the empty test cavity (without the sample) and tightened the fixing screws to verify accuracy. Finally, inserted the sample into the test cavity and started the tests. Note that different frequency bands require specific test fixtures and sample sizes. All composites were cut into films to fit the test molds. For the frequency of 1.13–1.73 GHz, The samples were cut to size of 82.4 mm × 164.8 mm × 1 mm. For the frequency of 1.72–2.61 GHz, The samples were cut to size of 54.46 mm × 108.92 mm × 1 mm. For the frequency of 2.60–3.95 GHz, The samples were cut to size of 33.89 mm × 71.84 mm × 1 mm. For the frequency of 3.94–5.99 GHz, The samples were cut to size of 22.0 mm × 47.52 mm × 1 mm. For the frequency of 5.38–8.17 GHz, The samples were cut to size of 15.75 mm × 34.70 mm × 1 mm. For the frequency of 8.2–12.4 GHz, The samples were cut to size of 22.9 mm × 10.2 mm × 1 mm. Then, all samples were fixed on the sample holder to be tested.

Based on the results collected from the tests, we have elaborated two theoretical theories, Schelkunoff theory and Calculation theory, to comprehensively evaluate the effectiveness of EMI shielding performance [31]. While the Calculation theory was intensively used by the researchers to assess the reflection and absorption within the materials, Schelkunoff electromagnetic wave transmission line theory was used to qualitatively explain how the EMW interacts with the surface of the composite material. This theory emphasizes the role of impedance mismatch in determining the proportion of reflection and initial absorption at the material surface.

The total EMI shielding effectiveness (${\text{SE}}_{{\text{T}}}^{*}$) for incident electromagnetic waves (EMWs) of the PPy@Fe^3+^/T-CFS-MC is calculated from the shielding reflection (${\text{ SE}}_{{\text{R}}}^{*}$) and shielding absorption (${\text{SE}}_{{\text{A}}}^{*}$), where ${\text{SE}}_{{\text{T}}}^{*}$ is the summary of absorption effectiveness (${\text{SE}}_{{\text{A}}}^{*}$), refection effectiveness (${\text{ SE}}_{{\text{R}}}^{*}$) and multiple refection effectiveness (${\text{ SE}}_{{\text{M}}}^{*}$). ${\text{ SE}}_{{\text{M}}}^{*}$ can be ignored when ${\text{SE}}_{{\text{T}}}^{*}$ ≥ 15 dB. The calculation equations as follows:

3 $$ {\text{SE}}_{{\text{R}}}^{*} = 168 - 10\lg \left( {\frac{{\mu_{r} f}}{{\sigma_{r} }}} \right) $$

4 $$ {\text{SE}}_{{\text{A}}}^{*} = 131t\sqrt {f\mu_{r} \sigma_{r} } $$

5 $$ {\text{SE}}_{{\text{T}}}^{*} = {\text{SE}}_{{\text{R}}}^{*} + {\text{SE}}_{{\text{A}}}^{*} + {\text{SE}}_{{\text{M}}}^{*} $$

where $\mu_{r}$ is the permeability of the material, $f$ is the frequency, $\sigma_{r}$ is the conductivity of the material, and $t{ }$ is the thickness of the material.

The total EMI shielding performance (SE_T_) of EMWs penetrate in the material is calculated from the scattering parameters (S_11_ and S_21_), where SE_T_ is the summary of absorption effectiveness (SE_A_), refection effectiveness (SE_R_) and multiple refection effectiveness (SE_M_). SE_M_ can be ignored when SE_T_ ≥ 15 dB. (SE_A_ represents the ability of the material to attenuate EMW that penetrate the shielding material due to absorption; SE_R_ represents the reduction in the incident EMW traveling into the shielding material resulting from reflection. SE_M_ represents multiple reflections of EMW inside materials.) The calculation equations as follows:

6 $$ {1} = {\text{R}} + {\text{A}} + {\text{T}} $$

7 $$ {\text{SE}}_{{\text{A}}} = - 10\log \left( {\frac{T}{1 - R}} \right) = - 10\log \left( {\frac{{\left| {S_{21} } \right|^{2} }}{{1 - \left| {S_{11} } \right|^{2} }}} \right) $$

8 $$ {\text{SE}}_{{\text{R}}} { } = - 10\log \left( {1 - {\text{R}}} \right) = - 10\log \left( {1 - |{\text{S}}_{11} |^{2} } \right) $$

9 $$ {\text{SE}}_{{\text{T}}} = {\text{SE}}_{{\text{A}}} + {\text{SE}}_{{\text{R}}} + {\text{SE}}_{{\text{M}}} $$

EMI Shielding efficiency (%) calculation formula as follows:

10 $$ {\text{EMI }}\;{\text{Shielding }}\;{\text{efficiency}}\,{ }\left( {\text{\% }} \right) = 100 - \left( {\frac{1}{{10^{{\frac{{{\text{SE}}}}{10}}} }}} \right) \times 100. $$

#### Electrochemical Performance Measurements

A three-electrode test system (the samples as the work electrode, Pt foil as the counter electrode and Hg/HgO as reference electrodes) was performed using electrochemical workstation (CHI660E, China) test the PPy@Fe^3+^/T-CFS-MC in the aqueous electrolyte of 1 M H_2_SO_4_. Cyclic voltammetry (CV), Galvanostatic charge discharge (GCD) and Electrochemical impedance spectroscopy (EIS), and capacitive cycle stability are the main test methods. The areal capacitance (Cs, F cm^−2^) of the electrode was calculated according to the discharge time of the GCD curve. The calculation formula is:

11 $$ {\text{Cs}} = \frac{1}{{{\text{vsV}}}}\int {{\text{idV}}} $$

where ***i*** (A) is the discharge current, and Δ*V* (V) refers to the change in voltage during the discharge time Δ*t* (s). *S* (cm^2^) represents the geometry areal of the samples.

Energy density (E, Wh cm^−2^), and power density (P, W cm^−2^) were calculated from

the discharge curves in GCD curves using the following equations:

12 $$ {\text{E}} = \frac{1}{2}{\text{CV}}^{2} $$

13 $$ {\text{P}} = {\text{E}}/{\text{t}} $$

where *V* is the voltage drop, and *t* (s) is the discharging time.

## Results and Discussion

### Surface Appearance

**Fig. 1:**
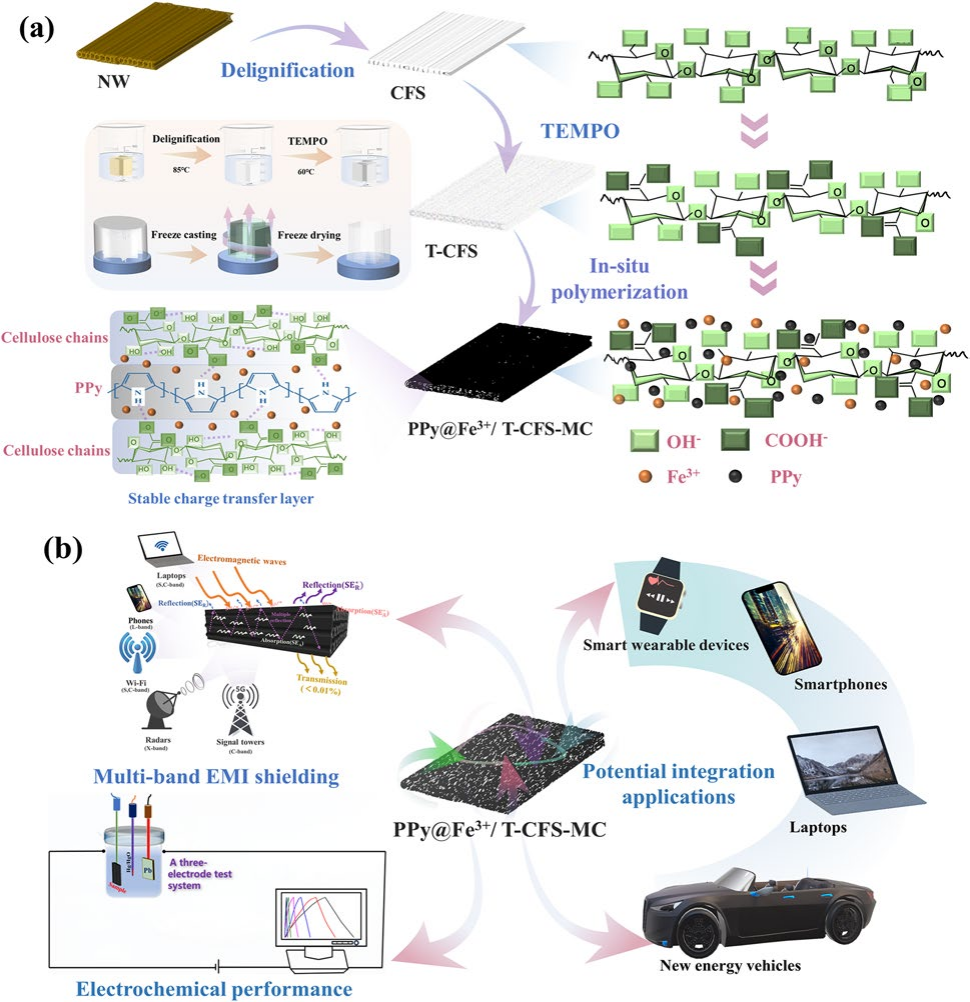
**a** Fabrication and mechanism diagram of the PPy@Fe^3+^/T-CFS-MC; **b** demonstration of multifunctional applications of the PPy@Fe^3+^/T-CFS-MC

**Fig. 2:**
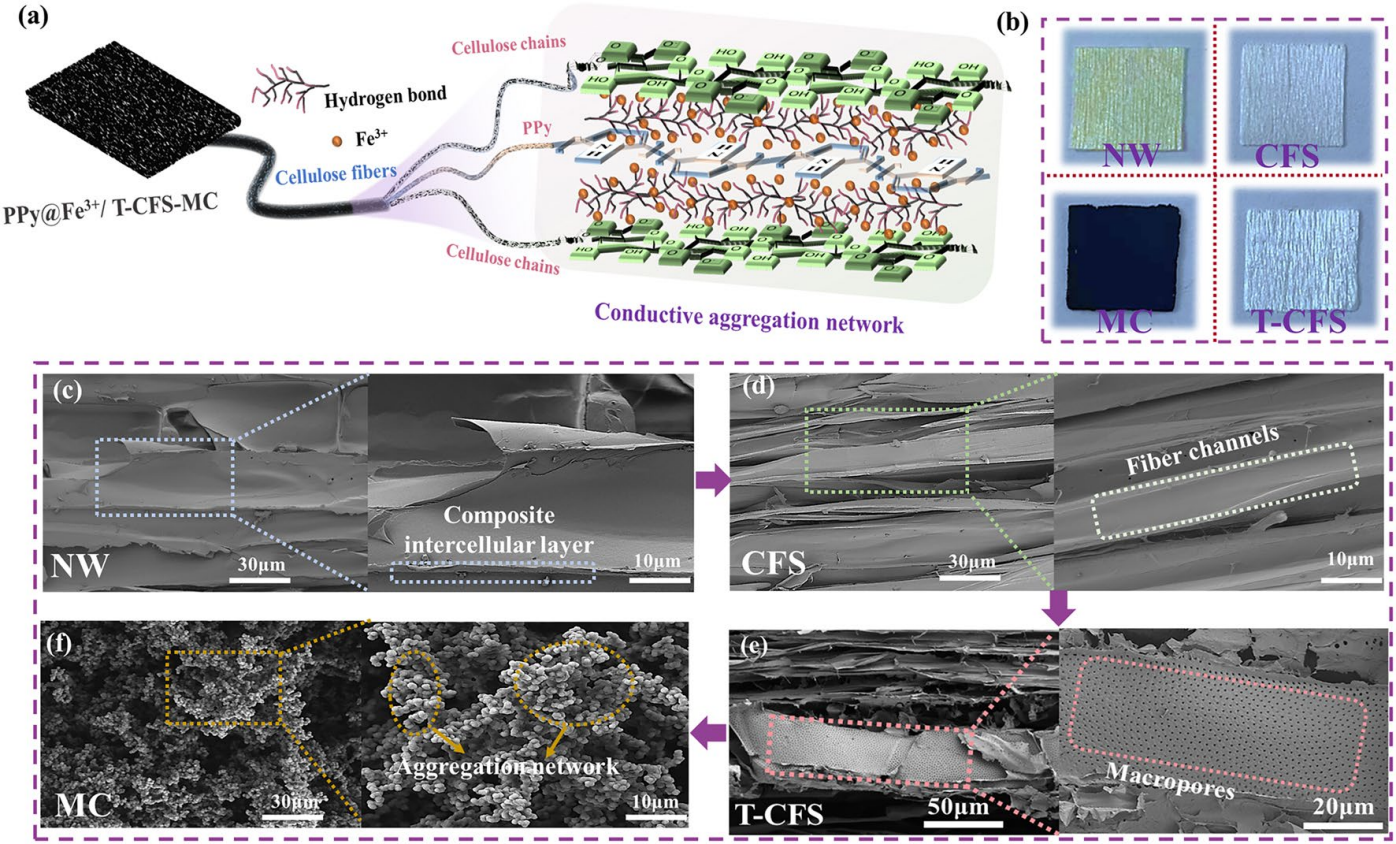
**a** Schematic diagram of the PPy@Fe^3+^/T-CFS-MC 3D conductive aggregation network; **b** Photographs of NW, CFS, T-CFS, and PPy@Fe^3+^/T-CFS-MC samples. The SEM images of **c** natural wood (NW), **d** cellulose fiber skeleton (CFS), **e** TEMPO-oxidized cellulose fiber skeleton (T-CFS), **f** TEMPO-oxidized cellulose fiber multifunctional composites (PPy@Fe^3+^/T-CFS-MC)

PPy@Fe^3+^/T-CFS-MC was fabricated employing a layered construction technique. Figure 1a elucidated the preparation process and mechanism. In detail, delignification was performed using NaClO_2_ under acidic conditions to obtain the CFS. Then, the T-CFS was produced through TEMPO oxidation. Finally, the PPy@Fe^3+^/T-CFS-MC was synthesized by in-situ oxidative polymerization of pyrrole in the presence of Fe^3+^. It was observed that the color changed from light yellow to white in CFS after the removal of lignin, and no visible changes on T-CFS compared to CFS, which is owing to the mild oxidation treatment (Fig. 2b). Notability, the dark blue color was observed on composites due to the successful polymerization of PPy.

Furthermore, the preparation of CFS was monitored by SEM images. As shown in Figs. 2c, d and S1a, b, the delignification treatment removed the composite intercellular layer between the fibers. The CNF were exposed, and the inherent cellulose nanochannel structure was preserved, indicating that the fiber structure remained intact. This enhancement in cell wall accessibility facilitated the chemical modification of the fibers. It was observed that rapid freezing with liquid nitrogen post TEMPO-mediated oxidation led to in-situ fibrillation and partial expansion of fibers while preserving the integrity of the microtubule array structure (Figs. 2e and S1c). In addition, the neatly arranged holes appeared on the fiber surface. These macropores were the pits on the tracheid of the wood structure. Without TEMPO treatment, the pits were partially open, limiting their accessibility (Fig. S1e). However, after the TEMPO oxidation process, these pits appeared to be fully opened. (Fig. 2e). This opening of the pits led to an increase in the specific surface area which enhanced the accessibility and exposure of the internal structure of the cellulose fibers.

Finally, the PPy@Fe^3+^/T-CFS-MC was obtained by adding pyrrole to the T-CFS for in-situ polymerization with ferric chloride as the oxidant. After TEMPO-mediated oxidation, the active sites and specific surface area of the fiber increased. Therefore, PPy was easily polymerized on the fiber surface, providing a higher active material load. As shown in Figs. 2f and S1d, the fiber surface was completely covered with a thick conductive layer, thereby ensuring the excellent electrochemical performance of the samples.

Figure 2a provides a detailed depiction of the conductive polymerization network mechanism of the sample in a three-dimensional state. It is well known that the surface of CNF contains abundant functional groups, and converting the hydroxyl groups on the surface of cellulose into carboxyl groups can effectively increase the surface charge density and is more conducive to the in-situ growth of conductive polymers. Hence, a mild modification system employing TEMPO/NaClO/NaClO_2_ (pH = 7) was devised to oxidize cellulose, thereby converting hydroxyl groups along the cellulose molecular chain into carboxyl groups. Specifically, T-CFS offered more active sites, allowing for polymerization of pyrrole with cellulose molecular chains aided by ferric ions. This led to the formation of a PPy charge transfer layer within the cellulose nanofibers, reducing resistive losses and enhancing charge transport, thereby creating a comprehensive three-dimensional conductive polymer network.

### Structure Properties

**Fig. 3:**
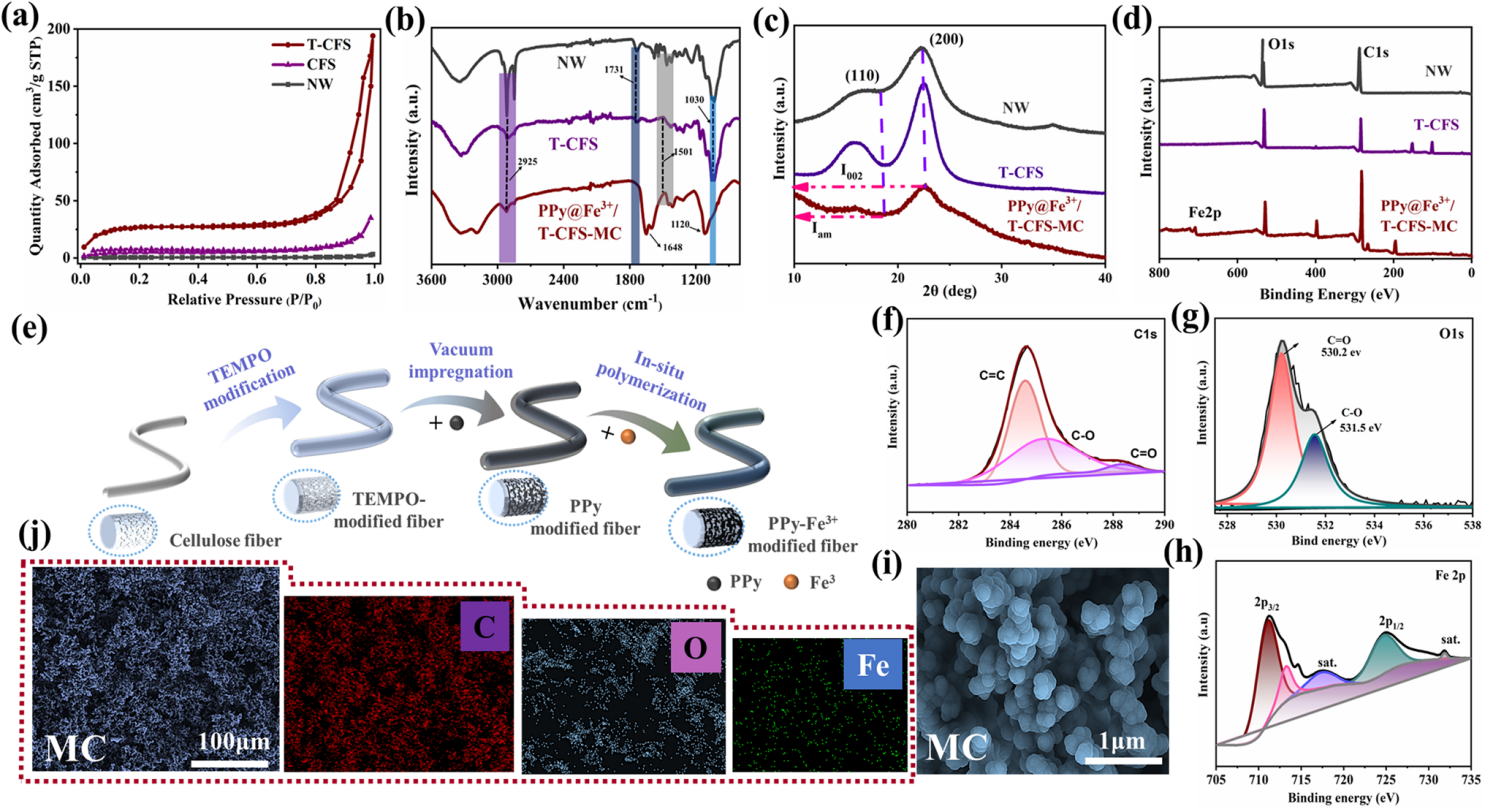
**a** N_2_ adsorption/desorption isotherms of the NW, CFS and T-CFS. **b** FTIR spectra of the NW, CFS, and PPy@Fe^3+^/T-CFS-MC samples. **c** XRD patterns of the NW, CFS, and PPy@Fe^3+^/T-CFS-MC samples. **d** XPS survey spectrum of the NW, T-CFS and PPy@Fe^3+^/T-CFS-MC. **e** Schematic diagram of fiber modification process. **f–h** C 1*s*, O 1*s*, and Fe 2*p* XPS spectra of PPy@Fe^3+^/T-CFS-MC. The SEM images **i** and C, O, and Fe-element EDX **j** of PPy@Fe^3+^/T-CFS-MC

To further analyze the relationship between internal structure and performance, the structure of the samples was characterized individually. First of all, the nitrogen adsorption/desorption isotherms were obtained to analyze the specific surface area of the samples quantitatively, and the results were plotted in Fig. 3a. As predicted, the nitrogen adsorption/desorption capacity of T-CFS samples was much higher than that of NW and CFS samples, and there was an obvious hysteresis loop between the adsorption curve and the desorption curve, indicating that there were many macropores which was consistent with the result that was observed in SEM (Fig. 2e) [39]. Meanwhile, the specific surface area was calculated by BET method, and NW had the smallest specific surface area, which was about 1.76 m^2^ g^−1^. And the specific surface area of CFS after delignification slightly increased to 19.2 m^2^ g^−1^. Noteably, the specific surface area of T-CFS was as high as 105.6 m^2^ g^−1^, which further proved that CFS had more active sites and a higher specific surface area after TEMPO-mediated oxidation.

In addition, as shown in the FT-IR spectra (Fig. 3b), the characteristic absorption peaks of lignin at around 1501 cm^−1^ (C=H stretching of aromatic rings) and hemicellulose at around 1731 cm^−1^ (C=O carbonyl stretching) on NW disappeared or weakened in the CFS spectrum, indicating that lignin and hemicellulose were effectively removed [40]. Besides, after TEMPO oxidation treatment of CFS, the primary hydroxyl at the C6 position was oxidized to carboxyl (-COOH), increasing the carboxyl content in the cellulose molecular structure. Therefore, after TEMPO oxidation, the C=O absorption peak around 1720 cm^−1^ was significantly enhanced, indicating an increase in the carboxyl content supporting and proving the successful hydroxyl esterification reaction [41]. Furthermore, the carboxylate content of both CFS (0.28 mmol g^−1^) and T-CFS (0.59 mmol g^−1^) was determined via acid–base titration, further confirming the generation of carboxyl groups (Fig. S2b). After in-situ polymerization treatment, compared to NW and T-CFS, the peak belonging to C–O–C stretching vibration was found blue shifted from 1030 to 1120 cm^−1^ and the peak intensity decreased in the spectrum of PPy@Fe^3+^/T-CFS-MC samples, owing to the PPy conjugation effectts occurred in the cellulose. Similarly, the spectrum of PPy@Fe^3+^/T-CFS-MC showed an obvious benzene ring stretching vibration peak at about 1648 cm^−1^, which indicated that PPy was in-situ polymerized on the T-CFS [42]. The mass loading of PPy on samples with and without TEMPO treatment were quantified. As shown in Fig. S3, after in-situ polymerization, the CFS had a weight increase of 190.7%; while the weight of T-CFS increased to 247.6% of its original weight. This higher loading of active substance was owing to the higher surface specific area and reactivity of T-CFS after TEMPO treatment.

Thermogravimetric analysis (TGA) and derivative thermogravimetric analysis (DTG) were employed to analyze the NW, CFS, T-CFS, and PPy@Fe^3+^/T-CFS-MC samples (Fig. S4). Firstly, upon comparison of the TG curves of NW, CFS, and T-CFS, several distinct stages were evident: In the initial stage (around 100–200 °C), a gradual mass decrease occured, likely attributed to the volatilization of free water. Subsequently, during the second stage (around 200–400 °C), a significant mass loss was observed, indicating rapid and substantial depletion of cellulose, hemicellulose, and lignin, the primary constituents of the sample. Moving into the third stage (around 400–800 °C), NW exhibited a residual mass after a gradual decrease, while CFS and T-CFS underwent complete mass loss following a slow decline. This discrepancy suggested that lignin in NW generate solid residual char post high-temperature exposure, whereas lignin within CFS and T-CFS is effectively eliminated. In contrast, it can be seen that the initial mass loss of the MC sample after drying is consistent with that of other samples, indicating that the adsorbed water is eliminated after drying. In the second stage (300–600 °C) where the cellulose was degraded, PPy@Fe^3+^/T-CFS-MC exhibited a slower degradation speed compared with the samples without PPy incorporation, which was owing to the robust thermal stability of PPy. The same trend was observed in the DTG curves, that MC had the highest peak temperature at 401.5 °C, indicating that the decomposition occurred at a higher temperature after the polymerization of PPy.

The crystalline structure of samples was subsequently studied using X-ray diffraction. As shown in Fig. 3c, all samples showed a typical signature of cellulose I structure with two broad crystalline reflections at around 16.4° and 22.2° in the 2θ range of 10–40° which indexed as (110) and (200), respectively [43, 44]. These results indicated that the crystal structure of cellulose in the constructed sample was not damaged and the overall structure of cellulose was preserved. In addition, the cellulose crystallinity of these samples was calculated according to the Siegel equation [45]:

14 $$ CrI = \left( {I_{200} - I_{am} } \right) \times 100 $$

where $I_{200}$ is the maximum intensity of the 200 lattice diffraction around 22.2° and $I_{am}$ is the intensity of amorphous diffraction at 18.2°.

According to the results that after delignification, cellulose crystallinity of the samples increased from 0.56 to 0.67 and then reduced to 0.39 after in-situ polymerization treatment. This decreased crystallinity of the PPy@Fe^3+^/T-CFS-MC was due to the swelling of wood fibers and the formation of conjugation and hydrogen bonding between cellulose chains and PPy molecular layers during the in-situ polymerization treatment.

Moreover, the elemental composition of the samples was analyzed using XPS measurement. From the survey spectrum in Fig. 3d, it can be seen that there was almost no change in the peak height of the C element and O element of NW and T-CFS, but the relative height of the C 1*s* peak of PPy@Fe^3+^/T-CFS-MC was greatly increased in comparison to NW and T-CFS. In contrast, the relative height of the O 1s peak was slightly reduced, which may be caused by the polymerization of PPy on the fibers. In addition, in the XPS spectrum of Fe 2*p* in Fig. 3h, the peaks concentrated at 711.3 and 724.8 eV were corresponding to Fe 2*p*_3/2_ and Fe 2*p*_1/2_, indicating that ferric ions were successfully introduced into the PPy@Fe^3+^/T-CFS-MC [46]. Similarly, the C 1*s* and O 1*s* XPS spectra were shown in Fig. 3f, g, the fitted binding energy peaks at 284.6, 286.3, 288.4, 530.2, and 531.5 eV were corresponding to C=C, C–O, C=O, C=O, and C–O respectively [47]. Additionally, Fig. 3e provided an intuitive description of the fiber modification process within the sample. Furthermore, Fig. 3i, j shows the elemental composition on the polymerized surface from EDX, which supports the successful introduction of ferric ions and pyrrole on the fiber. In conclusion, the above analysis results indicated that the PPy@Fe^3+^/T-CFS-MC has been successfully constructed.

Additionally, it is desirable to assess mechanical properties of the EMI shielding materials, the tensile strength of the NW, CFS, T-CFS, and PPy@Fe^3+^/T-CFS-MC samples was evaluated. As shown in Fig. S5, owing to the interconnected network formed together by the cellulose fibers, hemicellulose and lignin, NW exhibited the highest tensile strength and elongation at break at 18.7 MPa and 2.9%, respectively. The delignification process, in which most lignin and hemicellulose were removed, significantly reduced the tensile properties of the CFS. The tensile properties of T-CFS slightly declined after the mild TEMPO treatment due to the creation of the macropores on the fibers. Following the in-situ polymerization, the introduction of crosslinked polymer significantly enhanced the tensile strength of PPy@Fe^3+^/T-CFS-MC, reaching 15.1 MPa with elongation at break at 2%. These results demonstrated that the new EMI shielding materials possessed good mechanical strength.

### Electrical Conductivity and EMI Shielding Performance of the PPy@Fe^3+^/T-CFS-MC

**Fig. 4:**
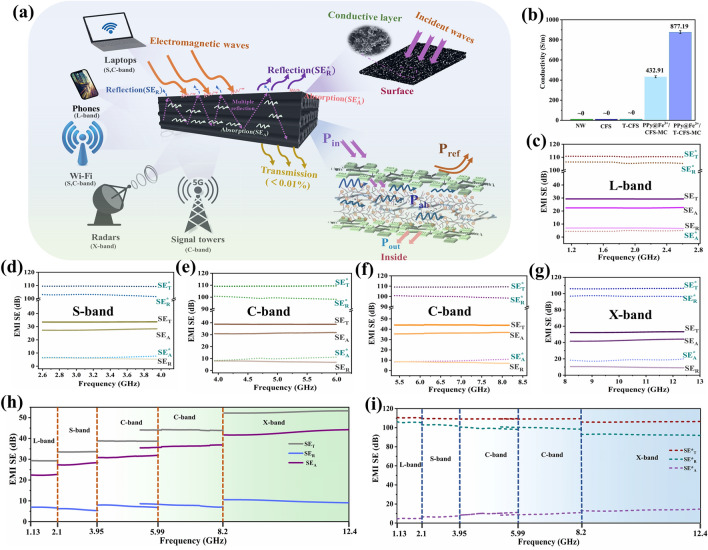
**a** Schematic diagram of the EMI shielding mechanism of PPy@Fe^3+^/T-CFS-MC. **b** Electrical conductivity of NW, CFS, T-CFS, PPy@Fe^3+^/CFS-MC, and PPy@Fe^3+^/T-CFS-MC samples. **c-g** Comparison of the EMI SE_T_, ${\text{SE}}_{{\text{T}}}^{*}$, SE_A_, ${\text{SE}}_{{\text{A}}}^{*}$, SE_R_ and ${\text{ SE}}_{{\text{R}}}^{*}$ of PPy@Fe^3+^/T-CFS-MC in the L, S, C, X-band, respectively. **h** EMI SE_A_, SE_R_ and SE_T_ of the PPy@Fe^3+^/T-CFS-MC in the frequency range of 1.13–12.4 GHz. **i** EMI ${\text{SE}}_{{\text{A}}}^{*}$, ${\text{ SE}}_{{\text{R}}}^{*} ,$ and ${\text{SE}}_{{\text{T}}}^{*}$ of the PPy@Fe^3+^/T-CFS-MC in the frequency range of 1.13–12.4 GHz

It is known that the conductivity of a material is positively correlated with its EMI shielding performance [48, 49]. Therefore, the electrical conductivity of the samples was measured first, and the results are shown in Fig. 4b. Prior to the introduction of PPy, all samples (NW, CFS, and T-CFS) exhibited nearly no conductivity as expected. In contrast, the sample with in-situ polymerization of CFS and pyrrole achieved high electrical conductivity, reaching 432.91 S m^−1^. This high conductivity was attributed to the formation of a complete conductive polymer network within the fiber network, which facilitated efficient electron transfer. Furthermore, PPy@Fe^3+^/T-CFS-MC, after mild TEMPO oxidation, showed higher electrical conductivity (877.19 S m⁻^1^) than PPy@Fe^3+^/CFS-MC. This enhancement was due to the increased active sites and the higher specific surface area on the fiber surface post-TEMPO oxidation, which enabled greater PPy mass loading to improve conductivity.

Unlike most reported EMI shielding materials that primarily target the X-band, EMI shielding performance of PPy@Fe^3+^/T-CFS-MC across the L, S, C, and X bands (1.13–12.4 GHz), covering the typical EMWs emitted by consumer electronic devices, was tested. This evaluation was conducted using a vector network analyzer and standard waveguide method. Because PPy@Fe^3+^/T-CFS-MC was a conductive composite with a 3D structure, its EMI shielding effect was studied in two stages: the reflection (${\text{SE}}_{{\text{R}}}^{*}$) and absorption (${\text{SE}}_{{\text{A}}}^{*}$) of incident EMW by the surface of the composite and the reflection (SE_R_) and absorption (SE_A_) of EMW within the composite [50].

Firstly, the EMI shielding effectiveness of the composites was evaluated at specific frequencies across different bands. As shown in Fig. S6, PPy@Fe^3+^/T-CFS-MC exhibited superior EMI shielding efficiency (99.99%) at various frequencies, with EMI SE greater than 100 dB. The total EMI values (${\text{SE}}_{{\text{T}}}^{*}$) shielded by the surface of the composite did not vary significantly at different frequencies, with the reflection being the predominant shielding effect. Once the EMW penetrated the surface, PPy@Fe^3+^/T-CFS-MC demonstrated better EMI shielding performance with higher frequency, and 99.99% of EMW was consumed within the composite, primarily by the internal absorption.

The detailed relationship between SE_T_, SE_A_, SE_R_, ${\text{SE}}_{{\text{T}}}^{*}$, ${\text{SE}}_{{\text{A}}}^{*}$, and ${\text{ SE}}_{{\text{R}}}^{*}$ in each band was investigated (Fig. 4c–g). Within the same frequency band, both EMI SE and the electromagnetic shielding value remained stable, indicating that the materials exhibited consistent EMI shielding effects. In general, ${\text{SE}}_{{\text{T}}}^{*}$ in each frequency band was much greater than SE_T_, demonstrating that most EMW was shielded by the surface of conductive composites. Moreover, ${\text{SE}}_{{\text{R}}}^{*}$ accounted for an average of approximately 95%, 93%, 91%, and 88% of the ${\text{SE}}_{{\text{T}}}^{*}$, respectively, while the SE_A_ accounted for an average of 76%, 82%, 80%, and 78% of the SE_T_, respectively. This indicated that in each frequency band, the majority of EMWs was reflected upon contact with the material surface (high ${\text{ SE}}_{{\text{R}}}^{*}$), and when EMWs penetrated into the interior of the materials, most of it was absorbed (high SE_A_). Figure 4h, j shows the EMI shielding effect for both incident and penetrated EMWs across the L- X bands, highlighting that shielding reflection was the dominant factor in the overall EMI shielding effect (${\text{SE}}_{{\text{R}}}^{*}$ ~ 100 dB).

The mechanism for the EMI shielding of PPy@Fe^3+^/T-CFS-MC is illustrated in Fig. 4a. Reflection, absorption, and multiple internal reflections occurred as the EMWs strike the surface of composites. Initially, ferric ions, PPy, and fibers formed a complete conductive network on the composite surface. Owing to the impedance mismatch between the air and the charge layer, EMWs were reflected by the surface of the PPy@Fe^3+^/T-CFS-MC via microwave, resulting in the reflection and dissipation of most EMWs, which accounted for the value of ${\text{SE}}_{{\text{R}}}^{*}$ [25, 51]. Subsequently, a portion of EMWs penetrated into the interior, generating a large amount of free charge on the charge transfer layer’s surface formed between PPy and fiber. Furthermore, the interaction of EMWs with the high electron density ferric ions, PPy and fibers generated ohmic losses, while the uneven distribution of charge caused dielectric loss, leading to thermal attenuation [52]. Finally, the porous structure of the entire skeleton facilitated multiple internal reflections of EMWs until they were completely absorbed, which accounted for the value of SE_A_. In summary, PPy@Fe^3+^/T-CFS-MC attenuated or eliminated EMWs through surface reflection, absorption, and multiple internal reflections, thereby achieving excellent EMI shielding performance.

Additionally, the EMI shielding properties of PPy@Fe^3+^/T-CFS-MC were compared to those of other reported EMI materials. As shown in Fig. S7 and summarized in Table S1, compared to other EMI shielding materials, such as metal-based, carbon-based, bio-based, and conductive polymer-based materials, the PPy@Fe^3+^/T-CFS-MC exhibited excellent EMI shielding performance in multiple bands, making it highly promising for application in the field of energy storage devices [9, 25, 51, 53–64].

While the current composite demonstrates strong EMI shielding effectiveness with a high reflection contribution, the materials with absorption-dominated behavior are preferable to prevent reflected electromagnetic waves from impacting nearby devices or environments. To enhance the EMI shielding performance of cellulose-PPy composite films via the absorption mechanism, some optimization strategies can be adopted, such as incorporating magnetic loss materials (e.g., Ni, Co) into the cellulose-PPy composite film, improving impedance matching between the material and free space by designing a multi-layer structure, with a cellulose fiber layer on the surface and a PPy layer on the inner side, and increasing the thickness of the cellulose-PPy composite films [65–67]. Overall, efficient absorption-dominated EMI shielding can be achieved by optimizing the composition, microstructure, and hierarchical design of cellulose-PPy composite films.

### Electrochemical Performance of the PPy@Fe^3+^/T-CFS-MC

The electrochemical performance of PPy@Fe^3+^/CFS-MC and PPy@Fe^3+^/T-CFS-MC were evaluated in a 1 M H_2_SO_4_ aqueous electrolyte through a three-electrode system, with Pt foil as the counter electrode and Hg/HgO as the reference electrode. The hydrolytic stability of the samples in a dilute sulfuric acid solution medium was first evaluated. As shown in Fig. S8a, the color of the solution remained unchanged after the samples were immersed for 48 h, and the sample retained 94.7% of its initial weight (Fig. S8c). Furthermore, the SEM image of the sample after immersion revealed no significant changes in its surface morphology, that the thick and continuous conductive polymer layers observed (Fig. S8b). These results demonstrated that the PPy@Fe^3+^/T-CFS-MC sample exhibited high stability in a dilute sulfuric acid medium, with no evidence of cellulose hydrolysis and polymer degradation.

**Fig. 5:**
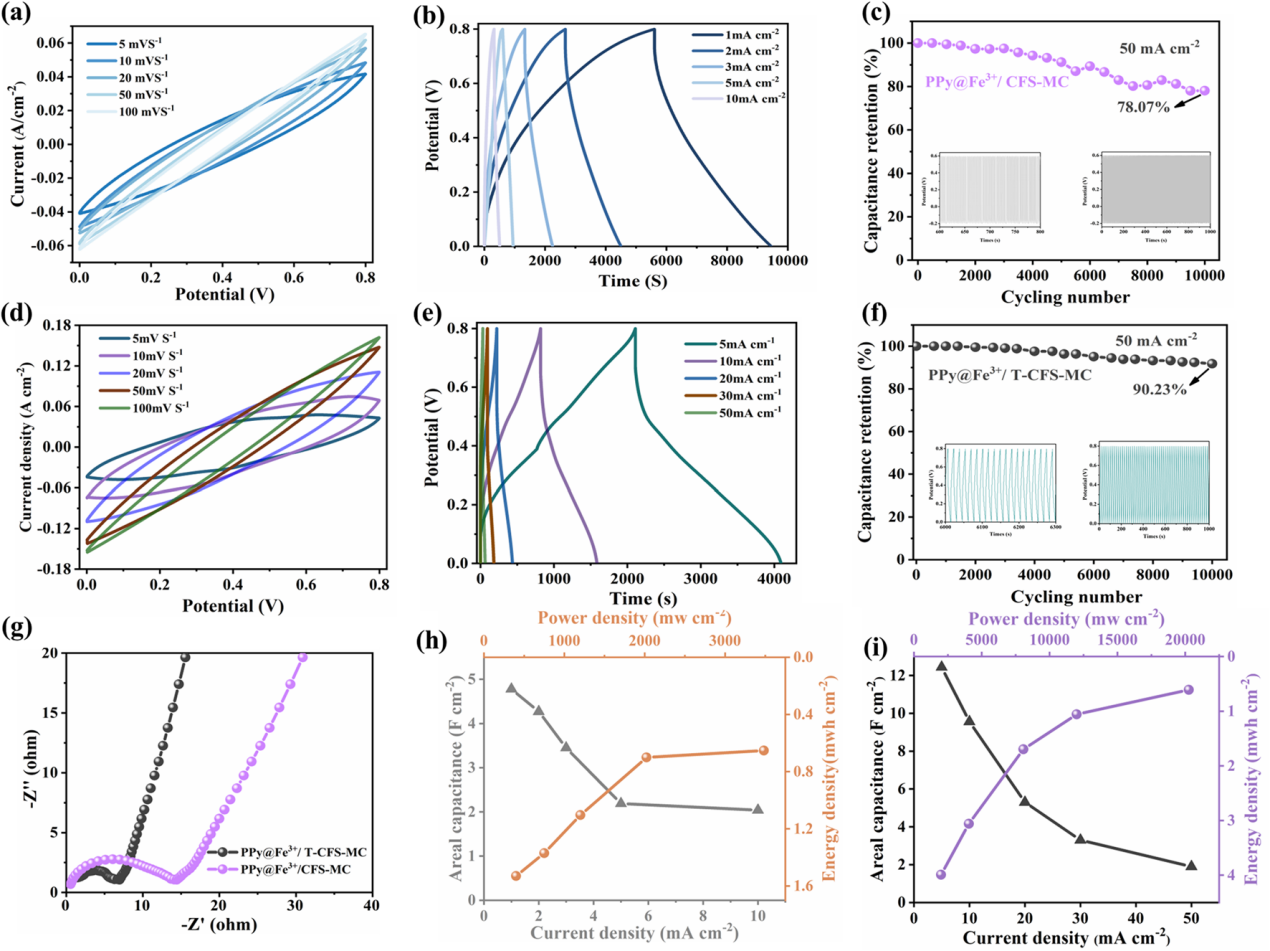
Comparison of electrochemical performances of PPy@Fe^3+^/CFS-MC and PPy@Fe^3+^/T-CFS-MC samples. PPy@Fe^3+^/CFS-MC samples: **a** CV curves at various scan rates, **b** charge–discharge profiles at different current densities, and **c** cycling stability at 50 mA cm^−2^ for 10,000 cycles. The PPy@Fe^3+^/T-CFS-MC samples: **d** CV curves at various scan rates, and **e** charge–discharge profiles at different current densities, and **f** cycling stability at 50 mA cm^−2^ for 10,000 cycles. **g** EIS plots of the samples after cycles. **h** The area capacitance at different current densities, and the areal energy/power density of the PPy@Fe^3+^/CFS-MC. **i** The area capacitance at different current densities and the areal energy/power density of the PPy@Fe^3+^/T-CFS-MC

In the following, its electrochemical performance was comprehensively evaluated. Figure 5a shows the CV curves of the PPy@Fe^3+^/CFS-MC samples collected at various scan rates in the potential range between 0 and 0.8 V. It can be observed that the CV curve of PPy@Fe^3+^/CFS-MC retained a fusiform shape at various scan rates with small, enclosed area, which did not achieve the desired capacitance performance. In contrast, the enclosed area of the CV curve for PPy@Fe^3+^/T-CFS-MC significantly increased at various scan rates (Figs. S9 and 5d). The highest current density reached 0.18 A cm^−2^, demonstrating excellent capacitive performance. This increase in capacitance was due to the higher mass load of PPy, which was derived from the TEMPO oxidation, allowing for an increased number of active sites on the fiber surface and enhanced specific surface area. The TEMPO modification also reduced ineffective resistance loss within the fiber, enabling efficient charge transport. Additionally, Fig. 5b, e displays the galvanostatic charge–discharge (GCD) curves of the PPy@Fe^3+^/CFS-MC and PPy@Fe^3+^/T-CFS-MC at various current densities. The GCD curve of PPy@Fe^3+^/CFS-MC did not exhibit an approximately isosceles triangle linear distribution. The discharge time was short at current densities of 1–10 mA cm^−1^, indicating that it could not support charge and discharge behavior under high current densities owing to the higher resistance and lower conductivity (as shown in Fig. 4a). In contrast, the GCD curve of the PPy@Fe^3+^/T-CFS-MC exhibited superior capacitive behavior at high current densities. This indicated the efficient charge transport within the PPy@Fe^3+^/T-CFS-MC because ions could be quickly and reversibly absorbed and desorbed on the surface. Furthermore, to evaluate the capacitance retention of the samples, capacitance cycling stability tests were conducted on both samples at a high current density of 50 mA cm^−2^. As shown in Fig. 5c, the PPy@Fe^3+^/CFS-MC exhibited a capacitance retention of 78.07% after 10,000 cycles, indicating a significant capacitance loss. In contrast, the PPy@Fe^3+^/T-CFS-MC exhibited improved capacitance retention of 90.27% after 10,000 cycles (Fig. 5f). This indicated that the TEMPO-oxidized CFS composites maintained their enhanced performance over the long-term. Concurrently, the increase in active sites on the fiber surface facilitated the complete polymerization of PPy within the fiber and the micropores formed the strong linkages between the fiber and the conductive polymer.

Additionally, EIS was used to compare the interfacial impedance of the two samples. Figure 5g showed distinct semicircular curves for the samples, indicating slightly higher resistance in the PPy@Fe^3+^/CFS-MC sample, which was the reason for the inferior results in charge–discharge and cyclic voltammetry tests. After the cycle stability test, the resistance of the samples further increased compared to that before the test. These changes was attributed to the loss of oxygen-containing functional groups during cycling, leading to the dissolution of part of the pseudocapacitance, which in turn increased resistance and attenuated capacitance [68]. The specific capacitances of the samples at various current densities were easily determined from the GCD curves. As depicted in Fig. 5h, i, the areal capacitance of PPy@Fe^3+^/CFS-MC was 2.19 F cm^−2^ at a current density of 5 mA cm^−2^. In comparison, at the same current density, the areal capacitance of PPy@Fe^3+^/T-CFS-MC reached 12.44 F cm^−2^, which was increased by nearly 6 times, revealing the excellent capacitive performance of PPy@Fe^3+^/T-CFS-MC. In addition, when the current density was 10 mA cm^−2^, the area capacitance of PPy@Fe^3+^/T-CFS-MC was 9.56 F cm^−2^. The areal capacitance still maintained at 76.85%, indicating that the composites had excellent stability within the relevant range of current densities. However, at a current density of 50 mA cm^−2^, the sample exhibited a dramatic decrease of areal capacitance to 1.9 F cm^−2^, with a significant voltage drop. This was due to the limitation of the material where charge adsorption/desorption could not be fully completed at high current densities.

Energy density and power density are significant factors for evaluating composites’ applicability. As shown in Fig. 5h, i, PPy@Fe^3+^/T-CFS-MC delivered the areal energy density of 3.99 mWh cm^−2^, corresponding to a power density of 2005 mW cm^−2^ at the current density of 5 mA cm^−2^. When the current density was 10 mA cm^−2^, the areal energy density and power density were 3.06 mWh cm^−2^ and 4047 mW cm^−2^, respectively. These numbers for PPy@Fe^3+^/T-CFS-MC were all higher than those of PPy@Fe^3+^/CFS-MC, representing a significant improvement. Notably, PPy@Fe^3+^/T-CFS-MC could also provide both high energy density (0.61 mWh cm^−2^) and high capacitance at high charge/discharge rates and a current density of 50 mA cm^−2^.

The electrochemical tests demonstrated the superior performance of PPy@Fe^3+^/T-CFS-MC. This outstanding electrochemical performance was attributed to several factors: (1) the unique 3D porous structure of the cellulose fiber enabled complete electrolyte penetration; (2) TEMPO oxidation-modified wood fiber achieved a higher specific surface area and more active sites, enhancing the active material loading during in situ polymerization of PPy; (3) PPy formed a stable charge transfer layer within the fiber network, reducing ineffective resistance loss and facilitating efficient charge transport. Therefore, these bio-based degradable composites have great potential for broad applications.

### Multifunctional Integrated Potential Applications of the PPy@Fe^3+^/T-CFS-MC

**Fig. 6:**
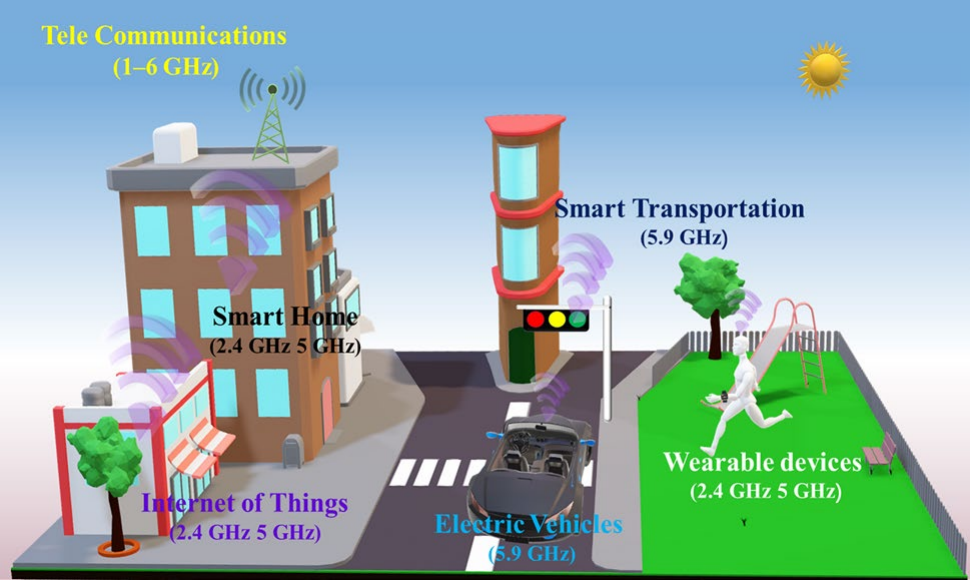
Schematic diagram of integrated material application scenarios

The PPy@Fe^3+^/T-CFS-MC integrates the functions of EMI shielding and energy storage. In practical applications, electronic devices often operate across multiple frequency bands, requiring materials that can shield against a broad spectrum of EMI. This multifunctionality allows the material to serve both as a multi-band EMI shielding component and an energy storage unit, making it highly valuable in 5G communications, wearable devices, electric vehicles, smart transportation, smart homes, etc. (Fig. 6). The development of this multifunctional integrated material not only helps to reduce the weight of equipment and simplify the design, but also meets the needs of modern technology for efficient, lightweight and multifunctional materials.

## Conclusions

A multifunctional cellulosic fiber composite (PPy@Fe^3+^/T-CFS-MC) was successfully constructed via in-situ polymerization, in which CFS was modified using a mild TEMPO oxidation system (TEMPO/NaClO/NaClO_2_, pH = 7). PPy@Fe^3+^/T-CFS-MC exhibited excellent electrochemical performance, high electrical conductivity (877.19 S m^−1^), and outstanding multi-band EMI shielding properties. It was found that most of the electromagnetic waves were first reflected directly, and the EMI values in the L, S, C, and X bands were over 100 dB, capable of effectively shielding most of the EMWs in our daily life and met the requirements for commercial EMI shielding materials. Meanwhile, benefiting from the high surface area (105.6 m^2^ g^−1^) and abundant active sites of T-CFS, the PPy@Fe^3+^/T-CFS-MC demonstrated a high areal specific capacitance of about 12.44 F cm^−2^ at 5 mA cm^−2^ and a high areal energy density of 3.99 mWh cm^−2^ corresponding to the power density of 2005 mW cm^−2^ with excellent stability of 90.23% after 10,000 cycles at 50 mA cm^−2^. The multifunctional cellulosic fiber-based composites constructed by the green and mild synthesis method possessed high electromagnetic shielding, high capacitance density, and excellent cycle stability. They could greatly benefit the development of more sustainable energy storage devices.

## Supplementary Information

Below is the link to the electronic supplementary material.

Supplementary file1 (DOCX 3261 KB)
