# Supplementary material for: Novel Cellulosic Fiber Composites with Integrated Multi-Band Electromagnetic Interference Shielding and Energy Storage Functionalities
Source: Nanomicro Lett. 2025 Jan 31;17:122. doi: 10.1007/s40820-025-01652-0 (PMC11785886; doi:10.1007/s40820-025-01652-0)
Supplement: Supplementary file 1 — Supplementary file1 (DOCX 3261 KB) [file 40820_2025_1652_MOESM1_ESM.docx]

Supporting Information for

**Novel Cellulosic Fiber Composites with Integrated Multi-Band Electromagnetic Interference Shielding and Energy Storage Functionalities**

Xuewen Han ^1, 2^, Cheng Hao ^2^, Yukang Peng ^1^, Han Yu ^1^, Tao Zhang ^1^, Haonan Zhang ^2,5^ Kaiwen Chen ^2.6^ Heyu Chen ^4^, Zhenxing Wang ^3^, Ning Yan ^2, *^ and Junwen Pu ^1, *^

^1^ Beijing Key Laboratory of Lignocellulosic Chemistry, College of Materials Science and Technology, Beijing Forestry University, Beijing 100083, P. R. China

^2^ Department of Chemical Engineering and Applied Chemistry, University of Toronto, Toronto, ON M5S3E5, Canada

^3^ Key Laboratory of Advanced Marine Materials, Ningbo Institute of Materials Technology and Engineering, Chinese Academy of Sciences, Ningbo 315201, P. R. China

^4^ College of Mechanical and Electronic Engineering, Northwest A&F University, Yangling, Shaanxi 712100, P. R. China

^5^ Jiangsu Provincial Key Lab of Sustainable Pulp and Paper Technology and Biomass Materials, Nanjing Forestry University, Nanjing 210037, P. R. China

^6^ Co-Innovation Center of Efficient Processing and Utilization of Forest Resources, Nanjing Forestry University, Nanjing 210037, P. R. China

*Corresponding authors. E-mail: [ning.yan@utoronto.ca](mailto:ning.yan@utoronto.ca) (Ning Yan); [jwpu@bjfu.edu.cn](mailto:jwpu@bjfu.edu.cn) (Junwen Pu)

**Supplementary Figures and Tables**


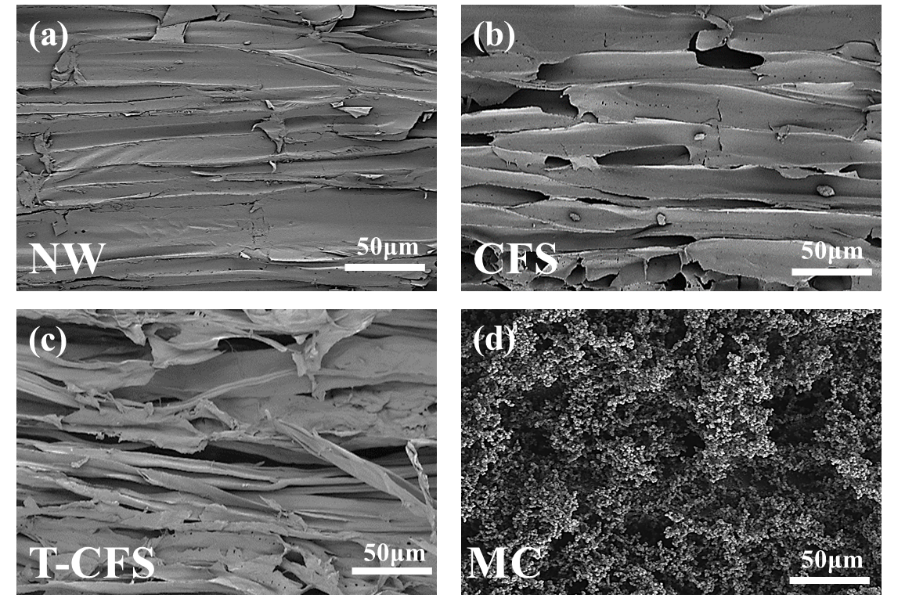

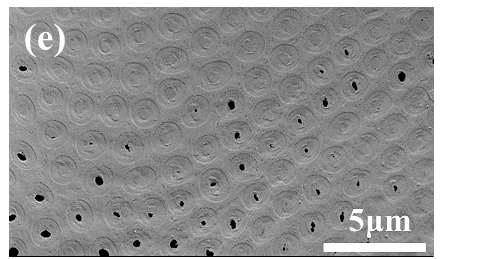


**CFS**

**Fig. S1** The SEM images of (**a**) natural wood (NW), (**b**) cellulose fiber skeleton (CFS); (**c**) TEMPO-oxidized cellulose fiber skeleton (T-CFS), (**d**) TEMPO-oxidized cellulose fiber multifunctional composites (PPy@Fe^3+^/ T-CFS-MC), (**e**) Tracheids structure on the surface of cellulose fibers


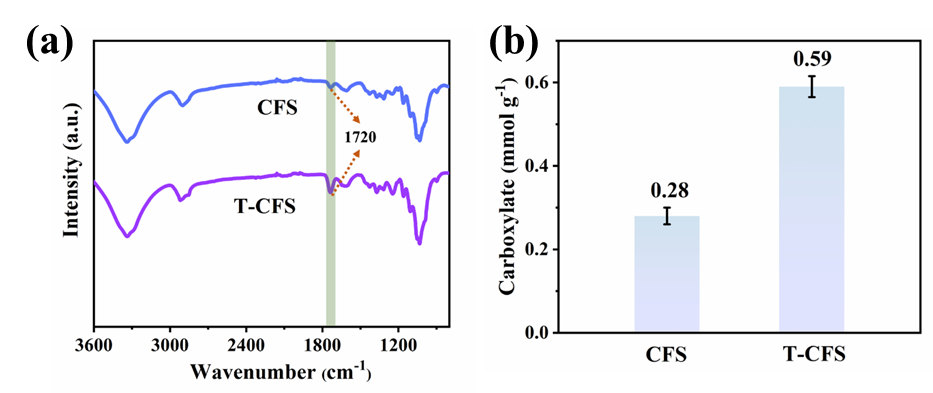


**Fig. S2** (**a**) FTIR spectra of samples before and after TEMPO oxidation; (**b**) Carboxylate content of CFS and T-CFS

**
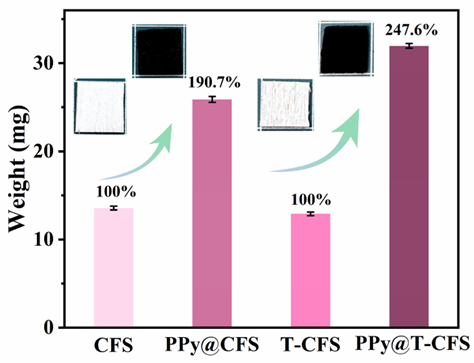
Fig. S3** Weight gain of PPy@Fe^3+^/ CFS-MC and PPy@Fe^3+^/T-CFS-MC samples


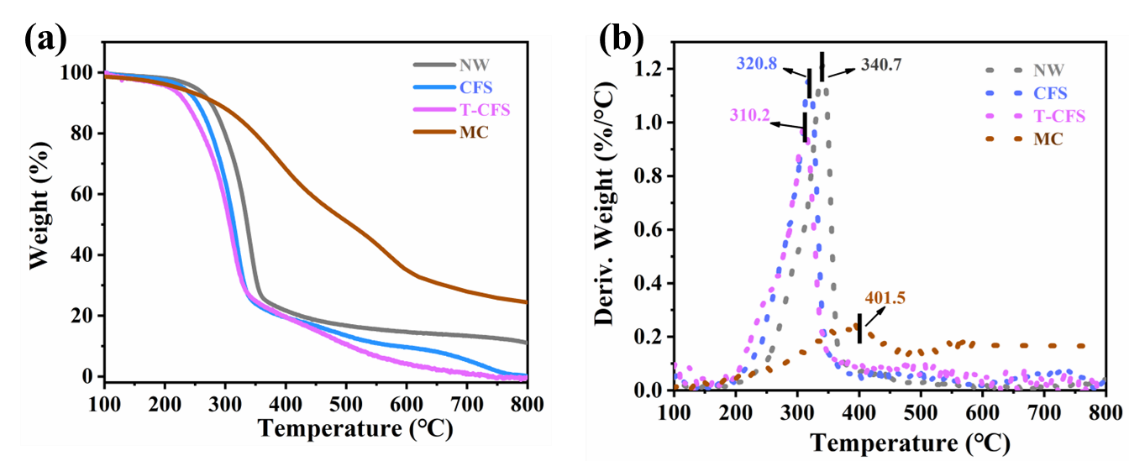


**Fig. S4** (**a**) TG and (**b**) DTG analysis of the NW, CFS, T-CFS and PPy@Fe^3+^/ T-CFS-MC samples


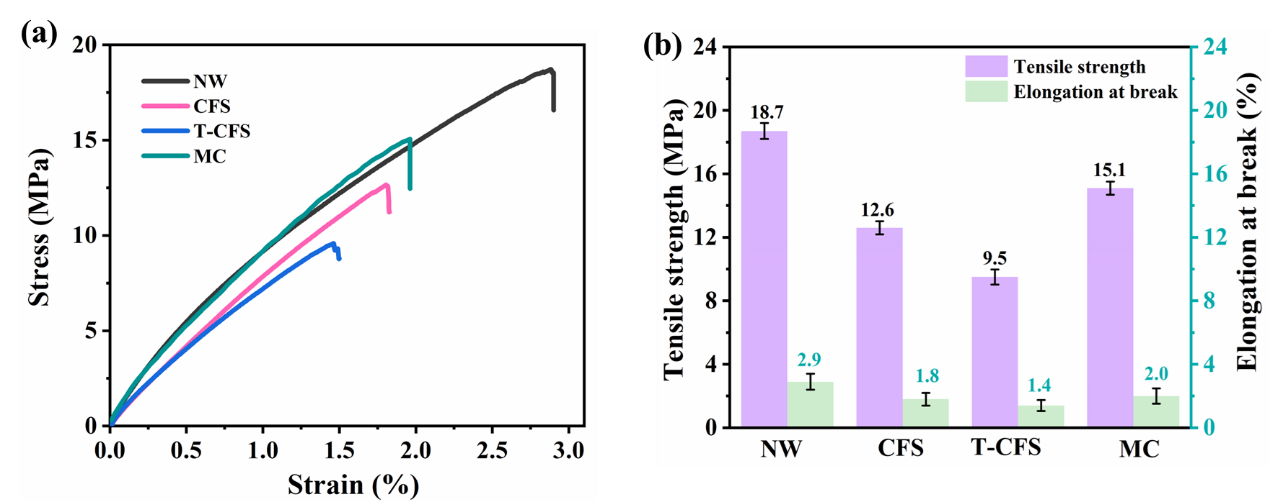


**Fig. S5** (**a**) Tensile stress-strain curves and (**b**) Maximum stress and strain of NW, CFS, T-CFS, MC samples

**
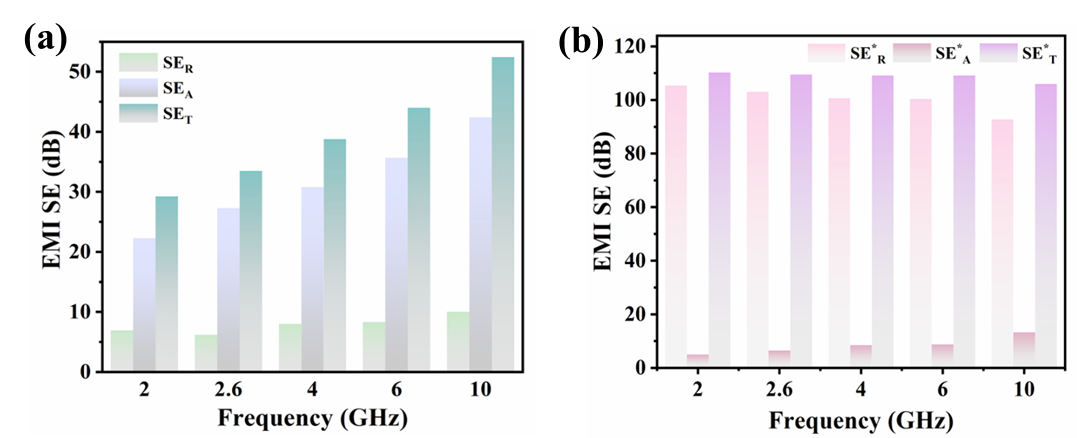
**

**Fig. S6** (**a**) The EMI SE_R_, SE_A_, SE_T_ of the PPy@Fe^3+^/T-CFS-MC at the frequency of 2, 2.6, 4, 6, 10 GHz, respectively. (**b**) The EMI $\text{ SE}_{\text{R}}^{*}$, $\text{SE}_{\text{A}}^{*}$, $\text{SE}_{\text{T}}^{*}$ of the PPy@Fe^3+^/T-CFS-MC at the frequency of 2, 2.6, 4, 6, 10 GHz, respectively

**
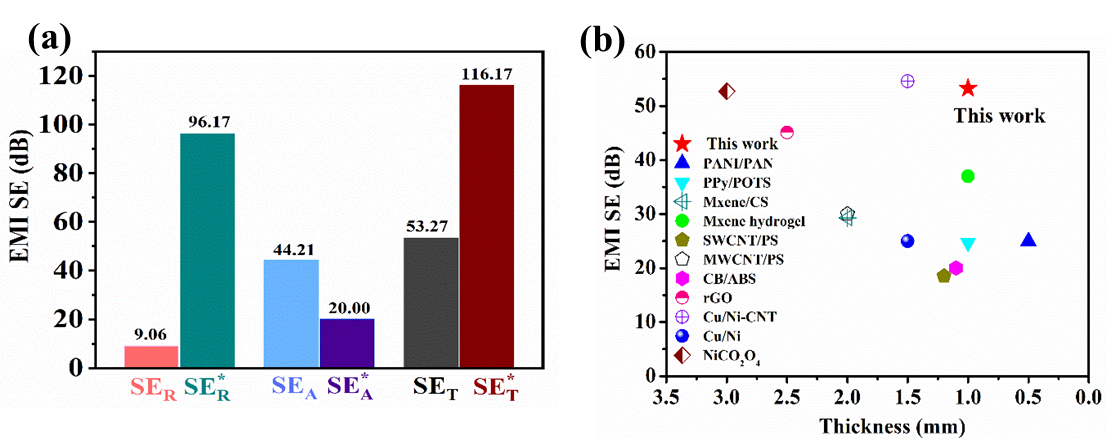
**

**Fig. S7** (**a**) Comparison of SE_T_, $\text{SE}_{\text{T}}^{*}$, SE_A_, $\text{SE}_{\text{A}}^{*}$, SE_R_ and$\text{ SE}_{\text{R}}^{*}$ of PPy@Fe^3+^/T-CFS-MC at the frequency of 12.4 GHz. (**b**) Comparison of EMI shielding performance (EMWs that only into the materials in the X-band) with other reported materials


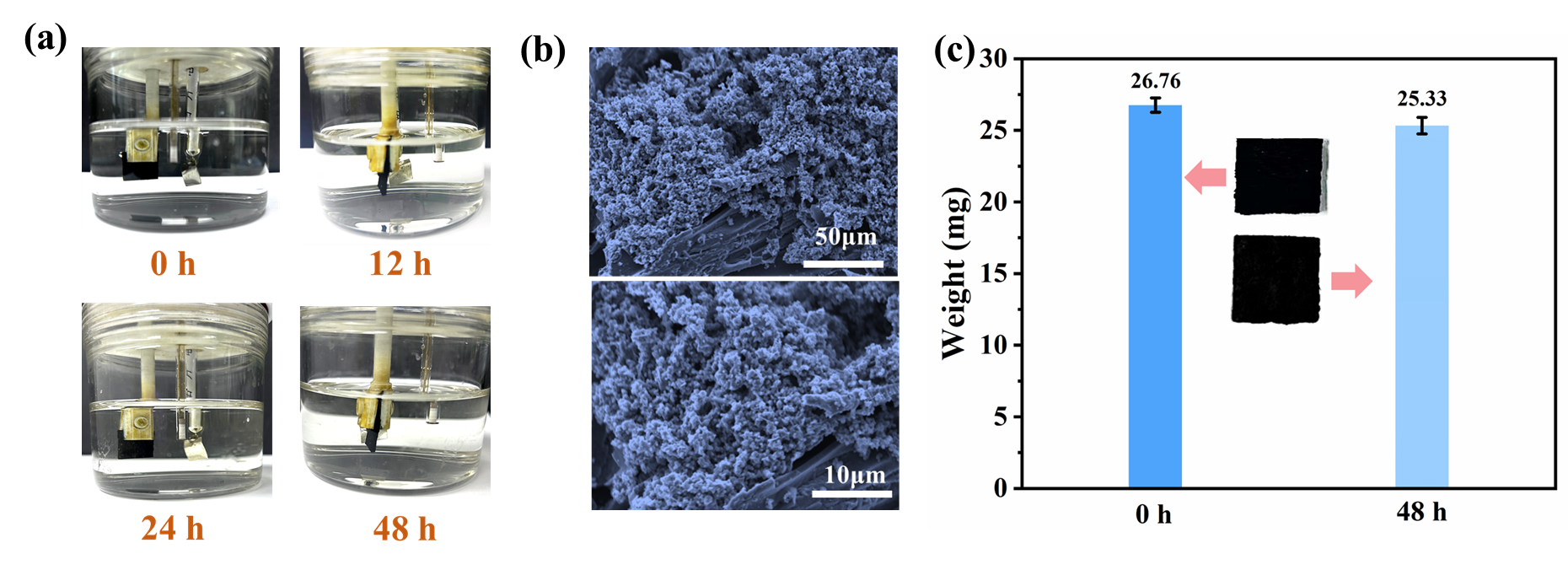


**Fig. S8** (**a**) The state change pictures of the PPy@Fe^3+^/T-CFS-MC sample placed in 1M sulfuric acid solution for 0, 12, 24, and 48 hours. (**b**) The SEM images of the MC sample after being placed in 1M sulfuric acid solution for 48 hours. (**c**) Weight change of the sample before and after being placed in 1M sulfuric acid solution

**
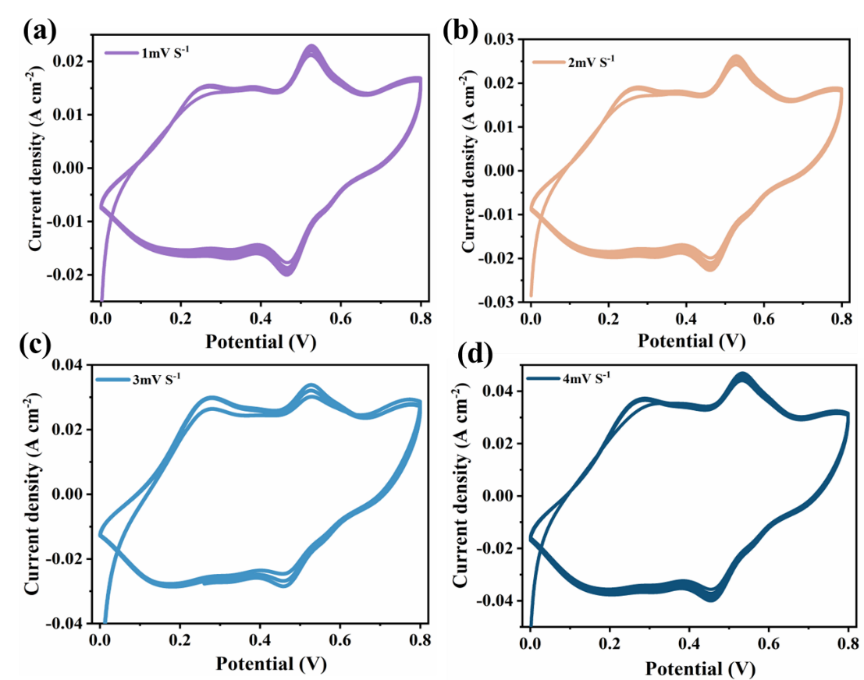
**

**Fig. S9** PPy@Fe^3+^/T-CFS-MC samples at CV curves at scan rates of (**a**) 1 mV S^-1^; (**b**) 2 mV S^-1^; (**c**) 3 mV S^-1^ and (**d**) 4 mV S^-1^

**Table S1** Comparison of the EMI shielding performance (EMWs that only into the material) of the PPy@Fe^3+^/T-CFS-MC with other reported materials [S1-S14]

| Type | Sample | Materials | Thickness  (mm) | L-band  SE (dB) | S-band  SE (dB) | C-band  SE (dB) | X-band  SE (dB) | Refs. |
| --- | --- | --- | --- | --- | --- | --- | --- | --- |
| **Metal-based** | 1 | NiCO_2_O_4_ | 3.0 | **/** | **/** | **/** | 52.72 | [S1] |
|  | 2 | Cu/Ni | 1.5 | **/** | **/** | **/** | 25 | [S7] |
|  | 3 | Cu/Ni-CNT | 1.5 | **/** | **/** | **/** | 54.6 | [S7] |
|  | 4 | NiFe_2_O_4_/rGO | 2 | **/** | **/** | 28.5 | / | [S13] |
| **Carbon-based** | 5 | rGO | 2.5 | **/** | **/** | **/** | 45.1 | [S6] |
|  | 6 | CB/ABS | 1.1 | **/** | **/** | **/** | 20 | [S11] |
|  | 7 | MWCNT/PS | 2 | / | / | / | 30 | [S10] |
|  | 8 | SWCNT/PS | 1.2 | **/** | **/** | **/** | 18.5 | [S9] |
|  | 9 | Mxene hydrogel | 1.0 | **/** | **/** | **/** | 37 | [S5] |
|  | 10 | EG flexible graphite | 0.127 | 55 | **/** | **/** | / | [S12] |
| **Bio-based** | 11 | CS/Mxene | 2 | / | / | / | 29.3 | [S8] |
|  | 12 | MWCNTs/ ANFs | 0.45 | / | / | / | 41.7 | [S2] |
|  | 13 | CNF/ Mxene | 3 | **/** | 47.9 | / | 52.6 | [S14] |
| **Polymer-based** | 14 | PPy/POTS | 1 | **/** | **/** | **/** | 24.7 | [S3] |
|  | 15 | PANI/PAN | 0.5 | **/** | **/** | **/** | 25 | [S4] |
| This work | 16 | PPy@Fe^3+^/T-CFS-MC | 1 | 29.3 | 33.6 | 44.2 | 53.27 |  |

CNT: carbon nanotubes; rGO: reduced graphene oxide; CB: carbon black; ABS: acrylonitrile butadiene styrene; SWCNT: single-walled carbon nanotubes; MWCNT: multi-walled carbon nanotubes; PS: polystyrene; EG: exfoliated graphite CS: cellulose scaffold; ANFs: aramid fibers; CNF: cellulose nanofibers; PPy: polypyrrole; POTS:1H, 1H, 2H, 2H-perfuorooctyltriethoxysilane; PANI: polyaniline; PAN: polyacrylonitrile.

**Supplementary References**

1. M. Zhang, C. Han, W.-Q. Cao, M.-S. Cao, H.-J. Yang et al., A nano-micro engineering nanofiber for electromagnetic absorber, green shielding and sensor. Nano-Micro Lett. **13**(1), 27 (2020). <https://doi.org/10.1007/s40820-020-00552-9>
2. L. Zou, C. Lan, S. Zhang, X. Zheng, Z. Xu et al., Near-instantaneously self-healing coating toward stable and durable electromagnetic interference shielding. Nano-Micro Lett. **13**(1), 190 (2021). <https://doi.org/10.1007/s40820-021-00709-0>
3. Z. Stempien, T. Rybicki, E. Rybicki, M. Kozanecki, M.I. Szynkowska, In-situ deposition of polyaniline and polypyrrole electroconductive layers on textile surfaces by the reactive ink-jet printing technique. Synthetic Met. **202**, 49-62 (2015). [https://doi.org/10.1016/j.synthmet.2015.01.027](https://doi.org/https://doi.org/10.1016/j.synthmet.2015.01.027)
4. Y. Yu, P. Yi, W. Xu, X. Sun, G. Deng et al., Environmentally tough and stretchable mxene organohydrogel with exceptionally enhanced electromagnetic interference shielding performances. Nano-Micro Lett. **14**(1), 77 (2022). <https://doi.org/10.1007/s40820-022-00819-3>
5. D.-X. Yan, H. Pang, B. Li, R. Vajtai, L. Xu et al., Structured reduced graphene oxide/polymer composites for ultra-efficient electromagnetic interference shielding. Adv. Funct. Mater. **25**(4), 559-566 (2015). [https://doi.org/10.1002/adfm.201403809](https://doi.org/https://doi.org/10.1002/adfm.201403809)
6. K. Ji, H. Zhao, J. Zhang, J. Chen, Z. Dai, Fabrication and electromagnetic interference shielding performance of open-cell foam of a Cu–Ni alloy integrated with cnts. Appl. Surf. Sci. **311**, 351-356 (2014). [https://doi.org/10.1016/j.apsusc.2014.05.067](https://doi.org/https://doi.org/10.1016/j.apsusc.2014.05.067)
7. Z.-x. Wang, X.-s. Han, Z.-j. Zhou, W.-y. Meng, X.-w. Han et al., Lightweight and elastic wood-derived composites for pressure sensing and electromagnetic interference shielding. Compos. Sci. Technol. **213**, 108931 (2021). [https://doi.org/10.1016/j.compscitech.2021.108931](https://doi.org/https://doi.org/10.1016/j.compscitech.2021.108931)
8. Y. Yang, M.C. Gupta, K.L. Dudley, R.W. Lawrence, Novel carbon nanotube−polystyrene foam composites for electromagnetic interference shielding. Nano Lett. **5**(11), 2131-2134 (2005). <https://doi.org/10.1021/nl051375r>
9. M. Arjmand, T. Apperley, M. Okoniewski, U. Sundararaj, Comparative study of electromagnetic interference shielding properties of injection molded versus compression molded multi-walled carbon nanotube/polystyrene composites. Carbon **50**(14), 5126-5134 (2012). [https://doi.org/10.1016/j.carbon.2012.06.053](https://doi.org/https://doi.org/10.1016/j.carbon.2012.06.053)
10. F. Jiang, Y.-L. Hsieh, Amphiphilic superabsorbent cellulose nanofibril aerogels. J Mater Chem. A **2**(18), 6337-6342 (2014). <https://doi.org/10.1039/C4TA00743C>
11. H. Guan, D.D.L. Chung, Radio-wave electrical conductivity and absorption-dominant interaction with radio wave of exfoliated-graphite-based flexible graphite, with relevance to electromagnetic shielding and antennas. Carbon **157**, 549-562 (2020). [https://doi.org/10.1016/j.carbon.2019.10.071](https://doi.org/https://doi.org/10.1016/j.carbon.2019.10.071)
12. R.S. Yadav, I. Kuřitka, J. Vilcakova, D. Skoda, P. Urbánek et al., Lightweight nife2o4-reduced graphene oxide-elastomer nanocomposite flexible sheet for electromagnetic interference shielding application. Compos. B Eng. **166**, 95-111 (2019). [https://doi.org/10.1016/j.compositesb.2018.11.069](https://doi.org/https://doi.org/10.1016/j.compositesb.2018.11.069)
13. B. Shan, Y. Wang, X. Ji, Y. Huang, Enhancing low-frequency microwave absorption through structural polarization modulation of mxenes. Nano-Micro Lett. **16**(1), 212 (2024). <https://doi.org/10.1007/s40820-024-01437-x>
14. H. Guo, Y. Li, Y. Ji, Y. Chen, K. Liu et al., Highly flexible carbon nanotubes/aramid nanofibers composite papers with ordered and layered structures for efficient electromagnetic interference shielding. Compos. Commun. **27,** 00879 (2021). [https://doi.org/10.1016/j.coco.2021.100879](https://doi.org/https://doi.org/10.1016/j.coco.2021.100879)
